# A European Society of Oncologic Imaging (ESOI) survey on the radiological assessment of response to oncologic treatments in clinical practice

**DOI:** 10.1186/s13244-023-01568-6

**Published:** 2023-12-20

**Authors:** Giovanni Cappello, Vittorio Romano, Emanuele Neri, Laure Fournier, Melvin D’Anastasi, Andrea Laghi, Giulia A. Zamboni, Regina G. H. Beets-Tan, Heinz-Peter Schlemmer, Daniele Regge

**Affiliations:** 1 Radiology Unit, Candiolo Cancer Institute, FPO-IRCCS, Str. Prov.le 142 km 3.95, 10060, Candiolo (Turin), Italy; 2 Department of Surgical Sciences, University of Turin, Turin, Italy; 3 Department of Translational Research, Academic Radiology, University of Pisa, 56124 Pisa, Italy; 4 Radiology Department, Hôpital Européen Georges Pompidou, AP-HP, Université de Paris, 20 Rue Leblanc, 75015 Paris, France; 5 Medical Imaging Department, Mater Dei Hospital, University of Malta, Msida, 2090 MSD Malta; 6 Department of Medical Surgical Sciences and Translational Medicine, Sapienza University of Rome, Sant’Andrea University Hospital, Via Di Grottarossa, 1035-1039, 00189 Rome, Italy; 7 Department of Diagnostics and Public Health, Institute of Radiology, University of Verona, Policlinico GB Rossi, P.Le LA Scuro 10, 37134 Verona, Italy; 8 Department of Radiology, The Netherlands Cancer Institute, P.O. Box 90203, 1006 BE Amsterdam, The Netherlands; 9 GROW School for Oncology and Developmental Biology, University of Maastricht, Maastricht, The Netherlands; 10 Department of Radiology, German Cancer Research Center (DKFZ), Im Neuenheimer Feld 280, 69120 Heidelberg, Germany; 11 Academic Radiology, Department of Translational Research on New Technologies in Medicine and Surgery, University of Pisa, Via Roma 67, Pisa, 56126 Italy

**Keywords:** Tumor assessment, Radiology reports, Standardization, RECIST 1.1, Clinical practice

## Abstract

**Objectives:**

To present the results of a survey on the assessment of treatment response with imaging in oncologic patient, in routine clinical practice. The survey was promoted by the European Society of Oncologic Imaging to gather information for the development of reporting models and recommendations.

**Methods:**

The survey was launched on the European Society of Oncologic Imaging website and was available for 3 weeks. It consisted of 5 sections, including 24 questions related to the following topics: demographic and professional information, methods for lesion measurement, how to deal with diminutive lesions, how to report baseline and follow-up examinations, which previous studies should be used for comparison, and role of RECIST 1.1 criteria in the daily clinical practice.

**Results:**

A total of 286 responses were received. Most responders followed the RECIST 1.1 recommendations for the measurement of target lesions and lymph nodes and for the assessment of tumor response. To assess response, 48.6% used previous and/or best response study in addition to baseline, 25.2% included the evaluation of all main time points, and 35% used as the reference only the previous study. A considerable number of responders used RECIST 1.1 criteria in daily clinical practice (41.6%) or thought that they should be always applied (60.8%).

**Conclusion:**

Since standardized criteria are mainly a prerogative of clinical trials, in daily routine, reporting strategies are left to radiologists and oncologists, which may issue local and diversified recommendations. The survey emphasizes the need for more generally applicable rules for response assessment in clinical practice.

**Critical relevance statement:**

Compared to clinical trials which use specific criteria to evaluate response to oncological treatments, the free narrative report usually adopted in daily clinical practice may lack clarity and useful information, and therefore, more structured approaches are needed.

**Key points:**

**·** Most radiologists consider standardized reporting strategies essential for an objective assessment of tumor response in clinical practice.

**·** Radiologists increasingly rely on RECIST 1.1 in their daily clinical practice.

**·** Treatment response evaluation should require a complete analysis of all imaging time points and not only of the last.

**Graphical Abstract:**

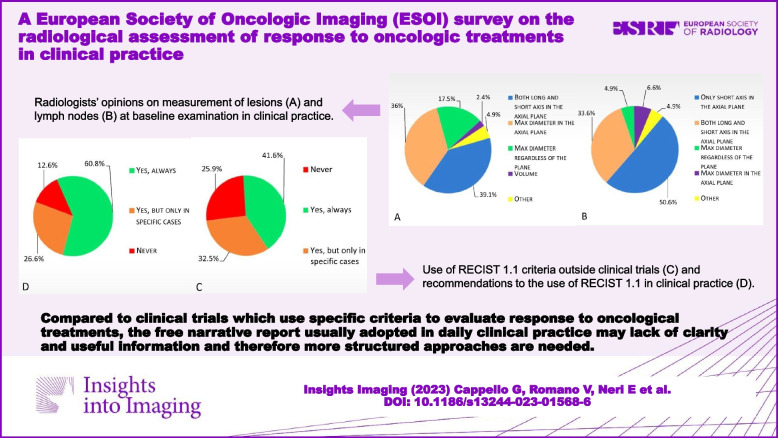

**Supplementary Information:**

The online version contains supplementary material available at 10.1186/s13244-023-01568-6.

## Introduction

Response evaluation criteria are crucial in the assessment of the efficacy of cancer drugs in clinical trials [1]. Four decades ago, at the dawn of cross-sectional imaging, the World Health Organization (WHO) introduced the first imaging criteria for the assessment of tumor burden, based on the sum of the products of diameters of the target lesions [2]. In 2000, the Response Evaluation Criteria In Solid Tumor (RECIST) working group published new guidelines, the RECIST version 1.0 [3], introducing new rules to better objectify the evaluation of the tumor burden, as the definitions of minimum size and the number of measurable lesions per organ. The new criteria also introduced unidimensional measurements bringing a simplification with respect to the WHO criteria [4]. A revised version, RECIST 1.1, that incorporates major changes such as a reduction in the number of lesions to assess, a new categorization of lymph nodes based on short axis, new recommendations for the assessment of progressive disease, and so on, was introduced in 2009 [5]. RECIST 1.1 criteria are based upon imaging modalities that are globally available and easily interpretable and are therefore widely used in clinical trials [6, 7].

Though intended for use in the clinical trial setting, oncologists increasingly rely on RECIST 1.1-based measurements for clinical management of patients also in daily clinical practice [8]. The main justifications are as follows: (1) the opportunity for a more standardized and structured approach to response assessment and (2) the increased clarity of the radiological report [9, 10]. Indeed, terms such as measurable disease, tumor burden, target lesions, and response categories are now part of the radiologist’s lexicon. However, in clinical practice, reporting strategies are mostly left to the local radiologist and oncologist, who may issue their own set of rules [11].

The need for a standardized approach, and of universally applicable rules for the assessment of response in daily routine, should be considered an important priority for the oncology and oncologic imaging community. The aim of this study was to investigate and compare the opinions and preferences of radiologists, with a dominant interest in oncologic imaging, on treatment response evaluation in clinical practice to gather information for the development of reporting models and recommendations.

### Materials and methods

To gather the opinions and preferences of radiologists, a survey was developed by an expert panel of members of the European Society of Oncologic Imaging (ESOI) board, composed of a radiology resident and two board-certified radiologists, the latter with more than 20 and 6 years of experience in oncologic imaging and expertise in tumor response assessment by RECIST 1.1 and other criteria.

The survey was conducted anonymously and was launched on the ESOI website (ww.esoi-society.org). ESOI members were reached on the same day by an email with a link to the survey; a week later, those who had not responded received a reminder and a final call was sent after 20 days.

The survey consisted of 5 sections with a total of 24 questions. In brief:

- The first section gathered demographic information (i.e., geographic distribution, age and site of main professional activity, and field of interest of participants).
- The second section focused on how to measure lesions and lymph nodes at the baseline examination and on how to deal with diminutive lesions.
- The third section was related to what to report on the baseline examination, which lesions to measure, how to evaluate non-measurable lesions, and which non-oncologic findings should be reported.
- The fourth section focused on reporting of follow-up examination and in particular which of the previous studies should be used as the comparator and how to compare previous findings.
- The fifth section included questions on the use of specific assessment criteria (mainly RECIST 1.1) in clinical practice. A focus is given on the medical imaging doctors’ practices and preferences including perceived advantages and disadvantages of using RECIST 1.1.

A web-based survey tool (Google Form, Mountain View, CA, USA) was used for the data collection. The results were downloaded and elaborated in Microsoft Excel format (Redmond, Washington, USA). Simple descriptive analyses and graphs were performed using Microsoft Excel 2018® (Microsoft Office, 2018). Proportions were compared using the “*N* − 1” chi-squared test [12]; a *p*-value < 0.05 was considered statistically significant.

## Results

Two hundred eighty-six completed forms were received and evaluated. The answers to all 19 questions related to Sects. 2–5 are reported in the supplementary material and demographics and professional information (Sect. 1, 5 questions) are summarized in Table 1.

**Table 1:** Demographic and professional information (286 responders)

	***No.* of responders (%)**
**Region**	
Europe	199 (69.6)
North and South America	38 (13.3)
Asia and Middle East	28 (9.8)
Africa	18 (6.3)
Australia and New Zealand	3 (1)
**Age**	
< 35	66 (23.1)
35–50	145 (50.7)
> 50	75 (26.2)
**Profession**	
Radiologist	244 (85.3)
Radiology Resident	25 (8.7)
Nuclear Medicine Physician	9 (3.1)
Other (surgeons, oncologists, imaging specialist)	8 (2.8)
**Working experience (in years)**	
< 5	58 (20.3)
5–10	57 (19.9)
> 10	171 (59.8)
**Place of work**	
University hospital	129 (45.1)
Research institute	18 (6.3)
Public hospital	74 (25.9)
Private hospital	56 (19.6)
Commercial company	5 (1.7)
Other	4 (1.4)

**Fig. 1:**
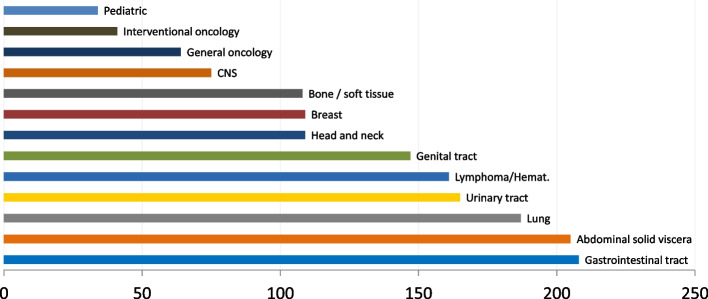
Main field(s) of oncologic imaging involvement of the responders. More than one response was allowed

In brief, most responders were from Europe (*n* = 199; 69.6%) and approximately half were aged between 35 and 50 (*n* = 145; 50.7%). Most responders had a working experience of more than 10 years (*n* = 171; 60%). The fields of interest of responders are summarized in Fig. 1.

### Baseline assessment

**Fig. 2:**
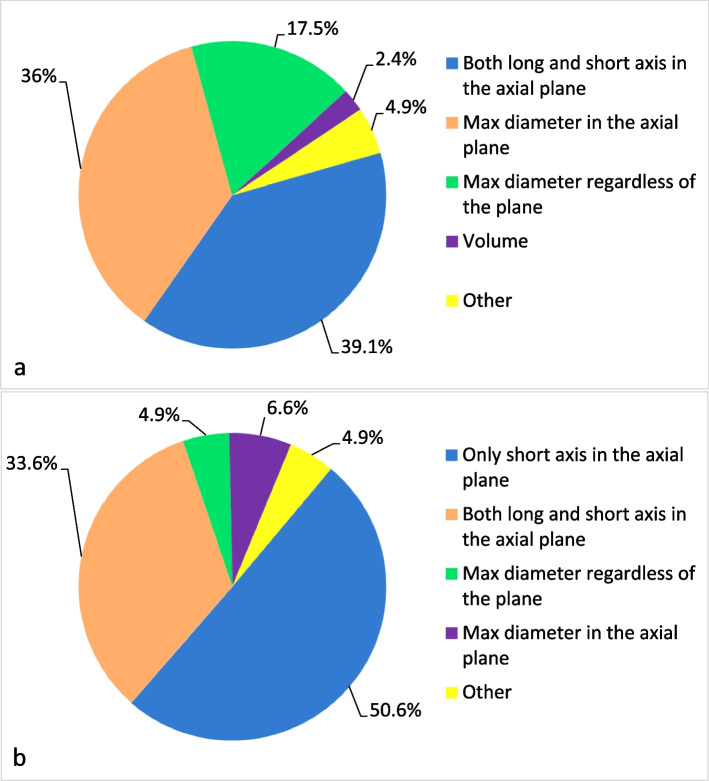
Radiologists’ opinions on measurement of lesions (**a**) and lymph nodes (**b**) at baseline in clinical practice

Figure 2 summarizes the preferred measurement criteria for organ lesions and lymph nodes. Mimicking the RECIST 1.1 criteria, most responders measured only the main lesions (182; 63.6%), preferably two per organ if present (132 of 182; 64.7%). Approximately half of the responders (145; 50.6%) reported measuring only the short axis of lymph nodes.

Non-measurable lesions (e.g., pleural effusion, abdominal fluid collection, peritoneal carcinomatosis) were mainly assessed qualitatively (*n* = 176; 61.5%); however, a minority of responders (*n* = 103; 36%) preferred a quantitative evaluation whenever possible.

Concerning diminutive lesions, i.e., lesions with a diameter below a certain predefined threshold, more than half of the responders replied that they did not measure lesions smaller than or equal to 5 mm (*n* = 72; 25.2% if ≤ 5 mm and *n* = 112; 39.2% if ≤ 3 mm) but would mention them in the report (*n* = 168; 58.7%).

Most responders (*n* = 179, 62.6%) affirmed that they would report all non-oncologic findings at the baseline examination, including benign and non-clinically significant ones (e.g., hepatic, or renal cysts), while a minority (*n* = 103; 36%) would report only the clinically significant findings.

According to the large majority of survey responders, tumor measurements were reported in the text of a narrative report (*n* = 231; 80.8%). Measurements were transferred through hyperlinks together with the images for 13.6% (*n* = 39) of responders, while 15.7% (*n* = 45) of responders adopted a structured report.

### Follow-up examination

**Fig. 3:**
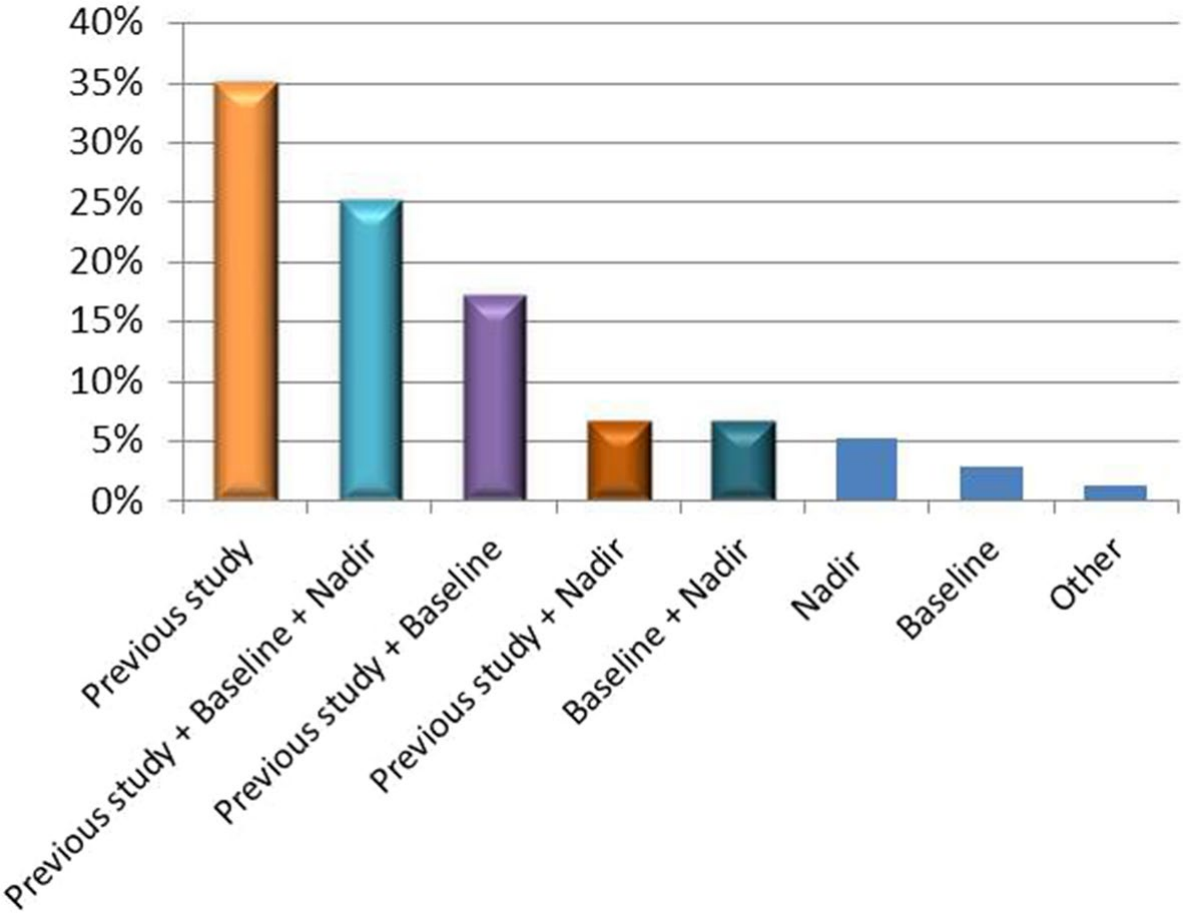
Percentage of which previous time point is used to compare the findings annotated in the baseline examination

Figure 3 shows responders’ opinions on which previous time point they use to compare the findings in real-world assessment. Of note, most responders (100 of 286; 35%) replied that they would compare findings with previous exam. In relation to measurable lesions, nearly all the responders report measuring the same lesions as in baseline (*n* = 249; 87.1%).

For non-measurable lesions, 46.2% (*n* = 132) of participants would continue evaluation only with qualitative assessment; conversely, 40.6% (*n* = 116) would perform an objective measurement of findings when feasible. With reference to non-oncologic imaging findings, almost half of the responders (*n* = 137; 47.9%) replied that they would report them only in case of significant changes, a summary sentence being recommended in all other cases (e.g., “all the other findings are unchanged”). Moreover, nearly all the responders (*n* = 245; 85.7%) conclude the report with their personal impression on the response to treatment.

### Use of RECIST 1.1 in clinical practice

Table 2 reports on the frequency of RECIST 1.1 use in real-world assessment. Overall, 74.1% of responders use RECIST 1.1 in their clinical practice either always or in specific cases. Of note, responders from research institutions use imaging criteria significantly less than responders from the remaining institutions. Table 3 summarizes the results on the opinion of responders on whether RECIST 1.1 should be used outside clinical trials. The overall rate of positive replies was 87.4%. Also, in this case, responders from research institution had a less favorable impression of the use of RECIST. Moreover, in reply to the specific question, 71% (*n* = 203) of responders, oncologists in their institution, consider response evaluation with RECIST 1.1 useful also in clinical practice.

**Table 2:** Responses to question 20 [Do you apply RECIST 1.1 criteria for response evaluation in clinical practice (not in clinical trials)?]. Results have been dichotomized according to geographic regions (Europe vs other countries), working experience (< 10 years vs > 10 years), and type of institutions (research facilities—university hospital and research institute vs other institutes). A *p*-value < 0.05 was considered statistically significant

	Never	Yes, always	Yes, but only in specific cases
Total no. of responses	74/286 (25.9%)	119/286 (41.6%)	93/286 (32.5%)
Europe	54/197 (27.4%)	75/197 (38.1%)	68/197 (34.5%)
Other	20/89 (22.5%)	44/89 (49.4%)	25/89 (28.1%)
*p*-value	0.18	0.08	0.32
Unexperienced	37/115 (32.2%)	42/115 (36.5%)	36/115 (31.3%)
Experienced	37/171 (21.7%)	77/171 (45%)	57/171 (33.3%)
*p*-value	0.06	0.13	0.72
Research facilities	48/148 (32.4%)	50/148 (33.8%)	50/148 (33.8%)
Other	26/138 (18.8%)	69/138 (50%)	43/138 (31.2%)
*p*-value	**0.01**	**0.006**	0.58

**Table 3:** Responses to question 21 (Do you think RECIST 1.1 criteria should be applied in clinical practice and not only in clinical trials?). Results have been dichotomized according to geographic regions (Europe vs other countries), working experience (< 10 years vs > 10 years), and type of institutions (research facilities—university hospital and research institute vs other institutes). A *p*-value < 0.05 was considered statistically significant

	Never	Yes, always	Yes, but only in specific cases
Total no. of responses	36/286 (12.6%)	174/286 (60.8%)	76/286 (26.6%)
Europe	26/197 (13.2%)	113/197 (57.4%)	58/197 (29.4%)
Other countries	10/89 (11.3%)	61/89 (68.5%)	18/89 (20.2%)
*p*-value	0.63	0.08	0.11
Unexperienced	17/115 (14.8%)	69/115 (60%)	29/115 (25.2%)
Experienced	19/171 (11.1%)	105/171 (61.4%)	47/171 (27.5%)
*p*-value	0.32	0.86	0.71
Research facilities	25/148 (16.9%)	82/148 (55.4%)	41/148 (27.7%)
Other institutes	11/138 (8%)	92/138 (66.7%)	35/138 (25.3%)
*p*-value	**0.02**	**0.04**	0.57

**Fig. 4:**
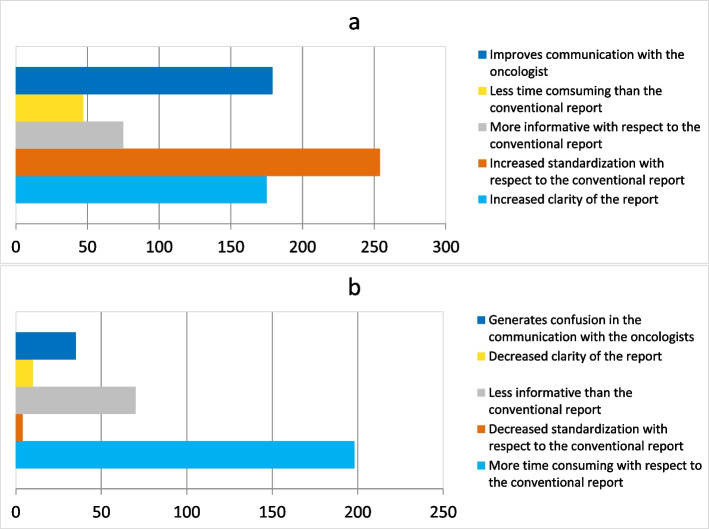
The charts show the perceived advantages (**a**) and disadvantages (**b**) of reporting with RECIST 1.1 criteria

Figure 4a and b report on the advantages and the disadvantages of reporting with RECIST 1.1 in clinical practice, respectively. Most responders consider the increased standardization with respect to the conventional report as the most important advantage. Conversely, most responders consider the use of RECIST 1.1 more time-consuming with respect to the narrative report.

## Discussion

Assessment of treatment response represents an important crossroad for the oncologic patient as it determines whether a specific drug, or ensemble of drugs, is effective or not. Within clinical trials, tumor response assessment relies primarily on the extent to which the sum of diameters of target lesions changes in time. Several imaging criteria have been developed for this purpose [13–18], being RECIST 1.1 [5] the most common. Opposite to the clinical trial environment where patients are carefully monitored, in daily clinical practice, the decision on whether to continue an oncologic treatment is left to the local multidisciplinary teams or to oncologists, who base their decision largely on their experience after gathering all useful clinical and imaging information. Cross-sectional imaging narrative reports usually provide fundamental information for decision-making. Unfortunately, narrative reports may lack standardization and clarity, and since recommendations are largely missing in this context, radiologists usually take a personal approach to reporting [19, 20]. Indeed, there is some evidence that narrative reports may not be as accurate as RECIST criteria in the assessment of response to treatment in clinical practice [21–23]. Feinderberg et al. showed that narrative reports were associated with an overestimation of treatment response in comparison to RECIST 1.1 among patients with complete response [21]. Schomburg et al. also compared the free text report with a response based on iRECIST criteria in 50 patients with metastatic renal cell carcinoma, finding only a moderate agreement between the two modalities (kappa 0.38 to 0.70), with new lesions frequently not recognized in free text [22]. These works underline the need for more standardized radiological criteria in daily clinical practice.

This survey was performed to gather information on radiologist’s practice in reporting cross-sectional imaging examinations in patients with advanced disease treated with cancer drugs in daily routine, to develop a common and shareable approach to the assessment of response to treatment. When preparing the questionnaire, the expert panel was aware that, since each cancer patient has a different story, conclusions drawn from its results would be difficult to generalize. This survey also aims to highlight the differences and criticalities of radiologists working in different institutions and with different professional backgrounds.

The first important observation is that most responders are inclined to follow RECIST 1.1 rules, with some notable exceptions. A slightly higher number of respondents (39.1% vs 36%) showed a preference for the measurement of two dimensions with respect to lesion maximum diameter only, even though the former is more time-consuming [24, 25]. We hypothesize that responders are more confident with measuring both long and short axis because they feel measures are more representative of the tumor, which often is characterized by an oblong and/or irregular shape. Only 7 respondents (2.4%) suggest measuring lesion volume, which is certainly more accurate, but not yet validated and time-consuming, unless a dedicated segmentation software is available [26]. Short axis diameter was considered the most reliable method to measure lymph nodes by half of the respondents. However, 33.6% of responders still prefer measuring both long and short axis of lymph nodes, probably because this method has been deemed more appropriate in some circumstances, for example in lymphoma assessment [15] or in predicting metastatic lymph nodes in gastric cancer [27]. Responders were not asked to define a cut-off measure for lymph nodes as size criteria are dependent on the lesion site. For example, inguinal lymph nodes may be considered pathological when the short axis is 15 mm or more while in other sites, e.g., mesorectum or mesenteric, the size cut-off is definitely smaller [28]. Moreover, morphology, shape, and borders may also be relevant in lymph node assessment [28].

Most radiologists measure lesions and lymph nodes on the axial plane, as recommended by RECIST 1.1. As for RECIST 1.1, most responders suggest measuring only the main lesions within each organ (63.6% of the cases) and a maximum of two lesions per organ (64.7%), following the selection criteria of target lesions, and to qualitatively evaluate diminutive and non-measurable findings, following the rules for non-target lesions.

According to RECIST 1.1 criteria, the baseline examination must be used as a comparator to define a stable, partial, or complete response to treatment and the nadir should be the reference to evaluate disease progression. Interestingly, in this survey, when deciding which of the previous studies should be considered as the comparator, responders expressed different opinions, some of which were controversial. Almost one third of responders affirmed that comparison should be performed only with the previous examination. In this regard, it must be noted that Weber et al. [11] developed a structured reporting concept for general follow-up assessment of cancer patients in clinical routine, based on RECIST 1.1 principles, but including only the prior tumor measurements, a limitation that the authors considered in their paper. It is the opinion of the authors that even in daily routine, a proper tumor response evaluation should require a careful comparison not only between the current and prior examination but also with older examinations, to avoid evaluation errors, as in the case of slowly growing lesions. One of the reasons why RECIST 1.1 response rules are difficult to apply in routine practice is that patient history is not always readily available and collecting clinical and imaging data is a hard and time-consuming process, especially when health electronic records are not readily accessible. Collaboration between radiologists and referring oncologists in this context is mandatory, and we recommend that controversial cases be discussed within a multidisciplinary framework.

In this survey, a substantial number of responders (41.6%) declare that they systematically use RECIST 1.1 criteria in clinical practice. Moreover, an even higher percentage (60.8%) believed that RECIST 1.1 should always be applied in clinical practice. This finding reflects the highly selected and motivated population of professionals that responded to this survey, mainly imaging specialists involved in oncologic reporting. However, interestingly, responders from research institutions use RECIST criteria less frequently than those working in other health facilities and believe they should be used in real-world assessment to a lesser extent. A non-negligible percentage of responders (32.5%) use RECIST 1.1 criteria in clinical practice only in specific cases. In free text answers, some responders affirmed that they prefer using RECIST 1.1 criteria in patients with mixed response, although the latter represents one of the well-known limitations of the criteria themselves. Other reasons that drive the radiologists to use RECIST 1.1 in daily routine are discrepancies between imaging and clinical data or cases with a high tumor burden and involving different organs, where a qualitative assessment can be difficult or misleading.

According to this survey, the main strengths of RECIST 1.1 are increased standardization, clarity, and improved communication with the oncologist. Opposite, the main concern of responders is the increased reporting time (68.5%). Of note, a minority of responders (16%) believe that the process is less time-consuming. Preference might depend on radiologists’ experience and on the availability of specialized software providing lesion identification, annotation, and allowing retrieval of target lesions from previous time point for comparison. These tools can create automatic reports with visual disease timelines and reduce errors by an automatic check when specific criteria are applied leading to a reduction of reporting time [29].

The main limitation of this study is represented by the specific target population that was addressed, i.e., members of the European Society of Oncologic Imaging, most of whom are imaging doctors with an interest in oncologic imaging. Indeed, general radiologists or oncologists might have different perspectives. Furthermore, responders are just a small proportion of the ESOI community, a fact that might have altered the results.

## Conclusion

To overcome the lack of rules, responders suggest either to use RECIST or personal criteria, usually a combination of unidimensional and bidimensional measurements of the most significant target lesions. Differently from RECIST, many responders suggest comparing the last time-point with the previous study, instead of baseline and nadir. A major concern of responders is that structured reporting is more time-consuming than a narrative report; this can be overcome by using specialized software. In conclusion, based on this survey, we believe it is important to define rules for the assessment of tumor response in clinical practice. The broader oncology community should take charge of their implementation.

### Supplementary Information

**Additional file 1. **Supplementary Tables.

## Data Availability

All data generated or analyzed during this study are included in this published article or in the supplementary material.

## Abbreviations

ESOI: European Society of Oncologic Imaging
RECIST: Response Evaluation Criteria in Solid Tumor
WHO: World Health Organization
