# Supplementary material for: A European Society of Oncologic Imaging (ESOI) survey on the radiological assessment of response to oncologic treatments in clinical practice
Source: Insights Imaging. 2023 Dec 20;14:220. doi: 10.1186/s13244-023-01568-6 (PMC10733253; doi:10.1186/s13244-023-01568-6)
Supplement: Supplementary file 1 — Additional file 1. Supplementary Tables. [file 13244_2023_1568_MOESM1_ESM.docx]

**A European Society of Oncologic Imaging (ESOI) survey on the radiological assessment of response to oncologic treatments in clinical practice**

**ELECTRONIC SUPPLEMENTARY MATERIAL**

**Table 1**: questions and answers of section 2 and 3 of the survey - reporting the baseline examination.

|  | **N° of responders (%)** |
| --- | --- |
| **How do you measure a lesion at baseline in your clinical practice?**  Both long and short axis on the axial plane  Maximum diameter on the axial plane  Maximum diameter regardless of the plane (axial, coronal, sagittal or oblique plane)  Volume  Other |  |
|  | 112 (39.1%)  103 (36%)  50 (17.5%)  7 (2.4%)  14 (4.9%) |
| **How do you measure a lymph node at baseline in your clinical practice?**  Only short axis in the axial plane  Both long and short axis on the axial plane  Maximum diameter regardless of the plane (axial, coronal, sagittal or oblique plane)  Maximum diameter on the axial plane  Other | 145 (50.6%)  96 (33.6%)  14 (4.9%)  19 (6.6%)  12 (4.9%) |
| **With reference to diminutive lesions, is there a size threshold below which you usually do not measure a lesion?**  3 mm or less  5 mm or less  10 mm or less  Size is not important  Other | 112 (39.2%)  72 (25.2%)  32 (11.2%)  52 (18.2%)  18 (6.3) |
| **How do you evaluate diminutive lesions at baseline in clinical practice?**  I always report them, but do not measure them  Size is not important, I always measure lesions  If they are too small I do not report them  Other | 168 (58.7%)  70 (24.5%)  28 (9.8%)  20 (7) |
| **At the baseline examination, which lesions do you measure?**  All the lesions, when feasible  Only the main lesions for each organ  Only the most important lesions, regardless of location/organ  Other | 61 (21.3%)  182 (63.6%)  33 (11.5)  10 (3.5) |
| **If You answered “Only the main lesions for each organ” to the previous question, how many lesions per organ do you measure?**  1  2  3 +  Other | 27 (13.2%)  132 (64.7%)  34 (16.7%)  11 (3.9) |
| **Do you evaluate non-measurable lesions at baseline (e.g, pleural effusion, abdominal fluid collection, peritoneal carcinomatosis)?**  Yes, I assess them qualitatively  No, I rather not report them if they are not measurable  I prefer to evaluate findings quantitatively, when feasible  Other | 176 (61.5%)  3 (1.0%)  103 (36%)  4 (1.4) |
| **Which non-oncologic findings should be pointed out in the baseline report?**  All findings, including benign and non-clinically significant findings  Only findings with a clinical relevance  Other | 179 (62.6)  103 (36)  4 (1.4) |

**Table 2**: questions and answers of section 4 of the survey - reporting the follow-up examination.

|  | **N° of responders (%)** |
| --- | --- |
| **When reporting the follow-up examination, which of the previous studies do you use as reference?**  Baseline solo  Previous study solo  Nadir or best response solo  Baseline + Previous  Baseline + Nadir or best response  Previous + Nadir or best response  Baseline + Previous + Nadir |  |
|  | 10 (3.5)  100 (35)  16 (5.6)  49 (17.1)  19 (6.6)  20 (7)  72 (25.2) |
| **During the follow-up evaluations, do you always measure the same lesions that were measured at baseline?**  Yes, when feasible  No, I prefer to measure all lesions when feasible  No, I prefer to measure well defined, easy to measure lesions even if they are different from those at baseline  No, I don’t measure lesions. Instead I give a general impression on response (e.g. mild/moderate/marked dimensional increase or decrease of the lesions) | 249 (87.1)  25 (8.7)  9 (3.1)    3 (1) |
| **During the follow-up examinations, how do you evaluate non-measurable lesions?**  I give a qualitative evaluation (e.g. “a mild/moderate/marked increase or decrease of the finding”)  I give both a qualitative assessment and a measurement, when feasible  I prefer to measure the findings, when feasible (e.g. the maximum thickness of pleural effusion)  Other | 132 (46.2)  116 (40.6)  35 (12.2)  3 (1) |
| **Which non-oncologic findings should be reported and described in follow-up examination?**  I report non-oncologic findings only if there is a significant variation, otherwise I write a summary sentence (e.g. ‘’all the other findings are unchanged’’)  Only findings with a clinical relevance should be reported (e.g. pulmonary embolism, kidney stones, AAA, bronchopneumonia, etc.)  All the findings should be reported even if already reported at baseline, including the benign and not clinically significant ones (e.g. hepatic cysts)  Other | 137 (47.9)  77 (26.9)  68 (23.8)  4 (1.4) |
| **In the conclusions of your report, do you give your final impressions on response (e.g. mild/moderate/marked dimensional increase or decrease of the lesions)?**  Yes  No  Other | 245 (85.7)  19 (6.6)  22 (7.7) |

**Table 3**: questions and answers of section 5 of the survey - assessment criteria for treatment response evaluation in clinical practice.

|  | **N° of responders (%)** |
| --- | --- |
| **Do you apply RECIST 1.1 criteria for response evaluation in clinical practice (not in clinical trials)?**  Yes, always  Yes, but only in specific cases  Never |  |
|  | 119 (41.6)  93 (32.5)  74 (25.9) |
| **Do you think RECIST 1.1 criteria should be applied in clinical practice and not only in clinical trials?**  Yes, always  Yes, but only in specific cases  Never | 174 (60.8)  76 (26.6)  36 (12.6) |
| **Do oncologists in your institution consider the assessment of the response to treatment with RECIST 1.1 criteria useful also in clinical practice?**  Yes  No  Other | 203 (71)  45 (15.7)  38 (13.3) |
| **In your opinion, what are the advantages of reporting with RECIST 1.1?**  Increased clarity of the report  Increased standardization with respect to the conventional report  More informative with respect to the conventional report  Less time consuming than the conventional report  Improves communication with the oncologist | 175  254  75  47  179 |
| **In your opinion, what are the disadvantages of reporting with RECIST 1.1?**  More time consuming with respect to the conventional report  Decreased standardization with respect to the conventional report  Less informative than the conventional report  Decreased clarity of the report  Generates confusion in the communication with the oncologists  None | 196  4  70  10  35  10 |
| **How do you present the tumor measurements in the radiological reports?**  In the text of a narrative report  Through hyperlinks to images or slices of the measured lesions in a narrative report  Using a structured report (including tables, graphs, etc...) | 231 (80.8)  39 (13.6)  45 (15.7) |

**Table 4**: free text answers to question 20 [(Do you apply RECIST 1.1 criteria for response evaluation in clinical practice (not in clinical trials)?], when responders replied “Yes, but only in specific cases “.

| In cases of targeted therapies or immunotherapy |
| --- |
| When it is mixed response or Oncologist asks |
| When I am asked by the oncologist |
| Clinical trials |
| When it is specifically requested by the oncologist |
| When asked by the clinician |
| I use RECIST "principles": how to measure, how to choose lesions, % change in sum of diameters as a marker of response assessment... but I feel that RECIST falls behind for clinical practice. It evaluates the treatment, not the patient. So I include many more nuances in may report, regarding lesions bur specially interpreting the findings and reporting toxicity. |
| If the clinician asks for it |
| Most of our patients are treated within clinical trials so if they have been previously treated within a trial I report the size of the target lesions in the previous trial. |
| Where requested |
| Only in clinical trials |
| By oncologist's request |
| When asked for by clinician |
| When there is discrepancy between clinic and imaging or in case of mixed response |
| If requested by the oncologist |
| Clinical trials |
| Modified RECIST, tailored to oncologist preference. |
| When it is not straightforward to use simple measurement |
| Patients selected for case discussion |
| Where relevant |
| Lymphoma |
| In cases with an expectation of response to treatment |
| Doubtful cases where some lesions are increased, other decreased. In these cases give also a RECIST1.1 evaluation seems more structured |
| Hospital practice |
| Measurable lesions, solid lesions |
| Only for patients in clinical trials |
| Only when requested by the referring clinician |
| Cases with have baseline image |
| When all follow up is made in our center |
| When requested by referrer, when response is not obvious by qualitative evaluation. |
| If requested by the oncologist |
| When it may change the patient's approach (based on clinical information) |
| I use RECIST 1.1 in almost all cancer follow ups, if appropriate. I do not use it in lymphoma. I do not use it if I do not understand the course of treatment and if I am unsure what the baseline examination is. |
| Usually on request of the oncologist, but with reservation, since RECIST criteria on the whole are too rigid to reflect the complexity of the clinical course outside the setting of a clinical trial. I also back up my assessment by discussing the assessment directly with the clinician on the phone |
| In case the patient is included in a clinical trial |
| When requested by the referring physician |
| Cases in which calculation is needed to allocate a category. |
| Solid Tumours or Lymphoma |
| When is feasible, if the response is heterogeneous then we prefer to leave it in a descriptive form |
| When the oncologist asked for it, but with a mark in the report |
| Clinical trials and when it is important for the course of treatment (eg: there is an increase, but not progression as for RECIST criteria) |
| When oncologists use and require RECIST 1.1 criteria |
| Solid organ lesions |
| When asked by clinical team (trial) |
| Only, if the clinical writs a clearly defined request, with important information as for example start of treatment |
| When there is a request from clinicians |
| PET/CT for restaging after therapy / for response to therapy |
| When surgeons ask |
| If applicable |
| When asked by the oncologist |
| When is needed for specific researches |
| Only in clinical trials |
| In lymphoma and follow up cases |
| When the oncologist asks |
| In solid tumours |
| When applicable to the tumour type |
| According to the demand of the oncologist |
| Malignancies which are treated parallels to study protocols |
| I do abdominal imaging. I evaluate cases by RECIST response categorization when I am sure that there are no other metastatic lesions (lung, CNS, etc.). In all cases I evaluate lesions size according RECIST rules but I do not write response category (for example: all lesions show significant decrease if they showed 30 or more percent reduction ) |
| Lung – liver metastases |
| If it was applied before (e.g., if I get to report the 4th follow-up scan and RECIST hasn't been used before, I will not use it either) |
| Rectum ca |
| Oncologist request |
| If it is specifically asked |
| If oncologist prefers it |
| Clinical trials context |
| When oncologist or patient asks |
| When the oncologist asks for it. I always keep RECIST 1.1 in mind when reporting, though, as the oncologists in our hospital are used to have this as a frame for response evaluation, also in clinical setting |
| Lymphoma, Lung, hepatobiliary, pancreas |
| When I'm asked to by oncologist |
| Neuroendocrine tumours, there is an agreement with our oncologists. |
| Lymphoma |
| Lung cancer |
| For common cancer: lung, gastric, rectal, cervical, breast, HCC. |
| When response in not straightforward, i.e., not obvious increase/decrease of lesions |
| When the oncologist asks and depending on the therapeutic... for GIST with imatinib I use CHOI criteria, for example |
| If it is required by the MDT treating team |
| Solid tumours |
| Research protocols |

**Table 5**: free text answers to question 21 (Do you think RECIST 1.1 criteria should be applied in clinical practice and not only in clinical trials?), when responders replied “Yes, but only in specific cases “.

| Chemotherapy |
| --- |
| When it is mixed response |
| Clinical trials |
| When the exam is performed to assess the changes under therapy, even though it is not a clinical trial |
| When there is an important number of lesion in different organs (high tumour burden exams) |
| Where RECIST performs well! |
| When we have no clear positive or negative response |
| In cases when clinically relevant and practically applicable |
| Guided by oncology team |
| Clinical trials |
| Solid malignancies |
| It generally helps me to evaluate difficult too many lesions or additional findings better and more confidently |
| When requested by the oncologist |
| Unfortunately, RECIST is extremely time consuming. The baseline is rarely done in our institution. Information regarding beginning of therapy is lacking. |
| Lymphoma |
| In control to treatment response |
| In cases of new treatment |
| Follow-up when RECIST was used in previous scan and when specified by the referral |
| Depending on type of therapy/tumour e.g., iRECIST or Choi, Deauville etc. |
| Ex as to use the short axis when measuring LN |
| Only if clear baseline and nadir study was defined. Also, if it will result in a change in management |
| When response is not obvious by qualitative evaluation |
| Too cumbersome and inaccurate |
| Whenever it may change the patient's approach |
| On request of the clinician, and with due reservation, and supported by direct communication with the clinician |
| If useful to solve doubt about clinical management |
| If there are clear target lesions |
| If the tumour response is not obvious, it can help to use formal (RECIST 1.1) criteria to support a clinical decision on changing/continuing/starting treatment |
| When better criteria (e.g., Choi or iRecist or LIRADS) are not available |
| Lymphomas |
| If the oncologist is not interested, why bother? Also, often too much discussion - clarification with consensus statement would be nice - even useable for general radiologists |
| Sometimes the answer is obvious without using criteria. Sometimes it is better to use other criteria |
| If applicable |
| As conclusion/report-summary for complex ones |
| When using a new treatment |
| When we cannot be sure about the response without use of RECIST 1.1 |
| In solid tumours |
| When applicable to the tumour type |
| E.g., when treatment reimbursement is based on RECIST results |
| Always when we can know for all metastatic lesions. Only way to be sure is there significant change we have to have some guidelines and orientation. |
| If I know the histology and what kind of therapy the patient has |
| When requested by oncologist |
| If referring doctors follow it strictly |
| Cases in which I know the baseline, the best response, and the chosen target lesions at baseline |
| Ref. 20a. It is a tricky question. We always keep RECIST1.1 as a thought of frame, but more flexible and nuanced in the clinical setting. And always leave the conclusion of PD etc. for the oncologist. E.g., summary: "general increase of tumour burden" / "general increased of size of lesions and new lung and liver metastasis" rather than "Progressive disease". |
| When measurement and comparison is feasible |
| When the ordering physician and radiologist have discussed the patient and there are clear lines of communication about therapy |
| For common cancer |
| When response is not clear or when asked by the oncologists |
| Kind of therapy |
| It depends on the tumour and on the treatment (example: GIST with imatinib - CHOI criteria) |
| When asked by oncologists |
